# Qualité de vie et vécu de la maladie, avant et après hystérectomie vaginale, chez les femmes admises au Centre Hospitalier Universitaire de Brazzaville

**DOI:** 10.11604/pamj.2016.25.79.10085

**Published:** 2016-10-17

**Authors:** Jean Alfred Mbongo, Alain Mouanga, Didace Massamba Miabaou, Aya Nzelie, Léon Hervé Iloki

**Affiliations:** 1 Service de Gynécologie Obstétrique, CHU de Brazzaville, BP 32, Brazzaville, Congo; 2 Service de Psychiatrie, CHU de Brazzaville, BP 32, Brazzaville, Congo; 3 Service Chirurgie Digestive, CHU de Brazzaville, BP32, Brazzaville, Congo

**Keywords:** Vécu, maladie, hystérectomie vaginale, Brazzaville, Congo, Subjective experience, disease, vaginal hysterectomy, Brazzaville Congo

## Abstract

Toute maladie est un mal en soi qu’il faut éradiquer car elle altère souvent de façon significative la qualité de la vie. L’hystérectomie vaginale est indiquée pour les patientes qui présentent certaines affections gynécologiques graves, elle est donc bénéfique mais, peut également avoir une répercussion néfaste sur la qualité de vie de la femme. Ainsi nous avons voulu explorer le vécu de la maladie et de l’hystérectomie vaginale (HV) des femmes avant et après l’intervention chirurgicale. Nous avons effectué une étude prospective qualitative, à recueil clinique sur une période de 12 mois; qui a concerné les femmes, ayant subi une hystérectomie vaginale. Celles n’ayant pas accepté de participer à l’étude, ou n’ayant pas de contact téléphonique n’ont pas été incluses. Pendant la maladie, le vécu des femmes a été: l’inconfort sexuel 26/40 (65%); les saignements génitaux 12/40 (30%); les douleurs pelviennes 13/40 (32,5%). En Post-opératoire, ont été noté les dyspareunies transitoires30/40 (75%) ; les céphalées secondaires à l’anesthésie 4/40 (10%). Le vécu psychologique a été dominé avant l’HV par la peur de la chirurgie chez toutes les patientes, les troubles du sommeil 38/40 (95%), l’angoisse 30 /40(75%), un sentiment de honte lié aux difficultés à accomplir l’acte sexuel en raison du prolapsus 26/40(65%) et/ ou en raison des saignements génitaux, dus au fibrome utérin 14/40(35%). Le sentiment de la perte de féminité était déclaré par 26/40 femmes porteuses de prolapsus utérin (65%), la modification de l’estime de soi 26/40 (65%). Ces appréciations subjectives ont été améliorées avec l’HV, contre balançant la perte de leur organe de reproduction. Aucune information n’a été donnée par les femmes à leurs proches et aux membres de la famille avant la chirurgie, traduisant ainsi leur sentiment de gène ou de honte. L’arrêt des symptômes a été observé dans tous les cas, même si dans un cas (1,25%) un nouveau signe au titre des complications (plaie rectale) a éténoté. Concernant l’activité sexuelle, tous les couples ont déclaré leur satisfaction après le traitement. Le vécu dramatique de la maladie et de l’hystérectomie vaginale avant, est nettement amélioré après l’intervention chirurgicale.

## Abstract (en)

Any illness is viewed as inherently evil, so it must be eradicated because it often significantly affect the quality of life. Vaginal hysterectomy is indicated in patients with some severe gynecological conditions; it is beneficial but can also have a detrimental impact on women’s quality of life. The aim of our study was to explore subjective experience of the disease and vaginal hysterectomy (VH) among women before and after surgery. We conducted a qualitative prospective study based on clinical data collection over a period of 12 months; it involved women who had undergone vaginal hysterectomy. Those who decided not to take part in this study or lack of phone contact were not enrolled. During their disease, women experienced: discomfort during sex 26/40 (65%); vaginal bleeding 12/40 (30%); 13/40 pelvic pain (32.5%). Postoperatively, transient dyspareunia 30/40 (75%); headache after anesthesia 4/40 (10%) were noted. Women’s psychological experience before VH was dominated by surgery fear in all patients, sleep disorders 38/40 (95%), anxiety 30/40 (75%), feelings of shame related to difficulty in performing the sexual intercourse because of prolapse 26/40 (65%) and/or because of genital bleeding due to uterine fibroids 14/40 (35%). The feeling of loss of femininity was reported in 26/40 women with uterine prolapse (65%), changes in self-esteem in 26/40 (65%). These subjective assessments were improved in women undergoing VH, offsetting the loss of their reproductive organ. No information was given by women to their loved ones and family members before surgery, reflecting their feeling of embarrassment or shame. The cessation of symptoms was observed in all cases, although in one case (1.25%) a new complication (rectal lesion) was noted. As regards sexual activity, all couples declared their satisfaction after treatment. Women’s dramatic experience of the disease and vaginal hysterectomy improved significantly after surgery.

## Introduction

La qualité de vie correspond pour l’Organisation Mondiale de la Santé à «… un large champ conceptuel, englobant de manière complexe la santé physique de la personne, son état psychologique, son niveau d’indépendance, ses relations Sociales, ses croyances personnelles et sa relation avec les spécificités de son environnement » (OMS, 1994). Le vécu correspond à l’ensemble subjectif des expériences, et des événements de la vie. Pour Vermersch [1], le vécu contient toutes les propriétés de la vie subjective y compris celles qui relève de l’accomplissement des actes (mentaux et matériel). La biographie de la personne est constituée par des éléments liés à son vécu.

L’hystérectomie vaginale est l’ablation chirurgicale de l’utérus en utilisant la voie naturelle sans ouverture de l’abdomen. Parfois, selon le cas, le col de l´utérus, les ovaires et les trompes de Fallope peuvent également être enlevés [2]. L’hystérectomie est, dans le monde occidental, l’intervention gynécologique la plus répandue, environ 70 000 hystérectomies sont pratiquées chaque année en France [3]. Le rôle de l’utérus dans la vie de la femme est, en dehors de sa fonction primordiale de support de la reproduction, particulièrement important dans la vision de son schéma corporel, dans sa sexualité et pour son psychisme. Il est donc licite de s’interroger sur les conséquences de l’hystérectomie sur la vie de la femme et notamment sur la qualité de sa vie sexuelle en particulier [3]. L’ablation de l’utérus, quelle qu’en soit l’indication, peut être mal vécue, en particulier parce queoutre la peur de l’intervention, des douleurs ou des complications viennent s’ajouter l’angoisse et/ou la honte de la perte de la féminité, de la modification de l’image corporelle. De plus, s’ajoute, la croyance que l’hystérectomie vaginale est associée à la ménopause, donc synonyme de vieillissement [4].

De même, la perte de l’organe les prive des menstruations et peut être vécue par certaines comme une perte de l’identité, de la désirabilité. Cependant l’hystérectomie vaginale peut les soulager de douleurs intenses ou de métrorragies abondantes; ce qui constitue souvent la motivation principale les poussant à accepter ce geste salvateur mais qui en ôtant leur utérus leur enlève l’attribut principal de la maternité. Le vécu d’une maladie gynécologique telle que le prolapsus utérin et le traitement subséquent du type hystérectomie vaginale; est une expérience très difficile à vivre par une femme; du, fait qu’elles font face aux complications inhérentes à d’une part à la maladie et d’autre part aux traitements, d’où altération importante de la qualité de leur vie. Aussi, nous nous sommes fixés pour objectif d’étudier la qualité de vie et le vécu de la maladie et de l’hystérectomie vaginale des femmes hospitalisées au CHU de Brazzaville avant et après l’intervention chirurgicale.

## Méthodes

**Figure 1:**
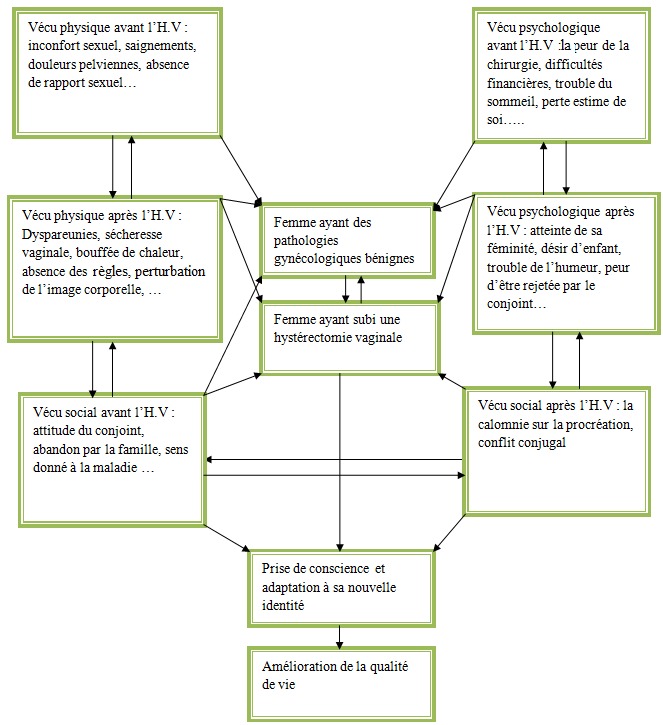
Schéma conceptuel, exprimant les problèmes qui influencent le vécu de la maladie et de l’hystérectomie vaginale par les femmes

Il s’agissait d’une étude qualitative et descriptive, avec méthode clinique, effectuée dans le Service de Gynécologie Obstétrique du CHU de Brazzaville durant une période de 12 mois, soit du 14 juillet 2014 au 14 juillet 2015. La population cible, a été constituée des femmes ayant subi une hystérectomie vaginale. Celles n’ayant pas accepté de participer à l’étude, ou n’ayant pas de contact téléphonique n’ont pas été incluses. L’échantillon a été constitué de façon non probabiliste, incluant toutes les femmes qui ont accepté de prendre part à l’étude en répondant à un questionnaire standardisé. Ainsi, et en tenant compte des critères d’inclusion 40 patientes ont été retenues pour l’étude. La Figure 1, représente le schéma conceptuel, exprimant les problèmes qui influencent le vécu de la maladie et de l’hystérectomie vaginale par les femmes. Les données ont été recueillies ont été analysées à l’aide du logiciel EPI-info.

## Résultats

### Caractéristiques sociodémographiques

L´âge moyen des femmes ayant subi une hystérectomie vaginale (FSHV) a été de44± 3ans avec des extrêmes de 32 à 65 ans; 67,9% d´entre elles étaient mariées, 25% étaient veuves et 7,1% étaient célibataires. La plupart des FSHV soit 80,7% avaient un niveau moyen, 19,3% avaient un niveau universitaire. L’occupation professionnelle a montré que 39,3% des FSHV étaient des commerçantes, 32,1% sans emploi et 28,6% étaient ménagères.

### Caractéristiques cliniques

Parité, 47% étaient Paucipares, 31%multipares, 10% grandes multipares et2% de nullipares. Indications de l’hystérectomie vaginale: prolapsus du col de l’utérus 26 cas, soit 65%, fibrome utérin 10 cas, soit 25%, dysplasies sévères du col utérin 4 cas, soit 10%. L’anesthésie pratiquée a été l’anesthésie générale 50% des cas, la rachianesthésie 48% et péridurale dans 2% des cas. Le Tableau 1, a mis en évidence la constance de l’inconfort sexuel chez les femmes présentant un prolapsus; celles souffrant de fibrome utérin se plaignaient de douleurs pelviennes, et de saignement génital avant l’hystérectomie vaginale; après l’intervention, elles se plaignaient de douleurs post-opératoires, et de céphalées (effets secondaires de la rachianesthésie). La description du vécu psychologique figure dans le Tableau 2. En effet, le prolapsus qui souvent était de 3^e^degré, et les saignements génitaux dus au fibrome utérin, entrainent une insatisfaction sexuelle pendant la maladie. De plus, après l’intervention, il est prescris un repos sexuel de 90 jours.

**Tableau 1:** Les éléments du vécu physique avant et après hystérectomie vaginale

	Avant HV		Apres HV	
	N=40	%	N=40	%
**Signes de la maladie**				
Douleurs	13	32,5	-	
Saignement génital	12	30	-	
Inconfort sexuel	26	65	-	
**Complications post opératoires**				
Douleurs	-		2	5
Céphalées	-		4	10
Plaie rectale	-		1	2,5
Dyspareunies	-		30	75
Ecoulement vaginal	-		1	2,5

^+^en rapport avec la reprise des rapports sexuels après hystérectomie vaginale

**Tableau 2:** Eléments du vécu psychologique avant et après hystérectomie vaginale

	Avant HV		Après HV	
	N=40	%	N=40	%
**Etat d’esprit**				
Trouble du sommeil	38	95	-	
Peur de la chirurgie	40	100	-	
Angoisse pour moyens de prise en charge	30	75	-	
**Rapports sexuels**				
Impossibilité en raison du saignement	14	35	-	
Difficultés en raison du prolapsus du col	26	65	-	
Durée d’attente avant la reprise écourtée	-		10	25
Dyspareunie à la reprise	-		40	100
Absence d’atteinte de l’orgasme	-		2	5
**Préparation à l’hystérectomie vaginale**				
Information sur les complications	-	-	-	
Soulagement de la souffrance	40	100	-	
Coût de la prise en charge élevé	40	100	-	
**Image corporelle**				
Perte de la féminité	26	65	-	
Perte d’organe de reproduction	-		40	100
Bouffées de chaleur	4	10	6	15
Sècheresse vaginale	8	20	10	25
Modification de l’estime de soi	26	65	-	-

A l’analyse du Tableau 3, concernant le vécu social des patientes, nous avons noté qu’elles n’ont pas informé leurs proches parents et leurs amis et relations sur leur état morbide, mais ces derniers avaient peur de la chirurgie et compatissaient avec elles dans les suites opératoires. Des cas d’infidélité du conjoint ont été signalés, mais la satisfaction sexuelle des couples après hystérectomie vaginale a été unanime dans l’ensemble.

**Tableau 3:** Analyse du vécu social avant et après hystérectomie vaginale

	Avant HV		Après HV	
Eléments	N=40	%	N=40	%
Information des proches sur la maladie	-			
Attitude de peur des proches pour la chirurgie	40	100	-	
Information des membres de la société	-		40	100
compassion des membres de la société après HV	-		40	100
**Vécu conjugal**				
-Infidélité du conjoint	4	10	6	15
-Satisfaction du couple aux rapports sexuels	24	60	40	100

## Discussion

La phase de manifestation de la maladie a été pour la plupart un moment délicat. Elles font face non seulement à la douleur mais aussi au saignement génital, et au désagrément du à l’extériorisation du col en rapport avec le prolapsus. Pour Farquhar CM et al [5], les problèmes gynécologiques comme les douleurs pelviennes, les saignements abondants et prolongés, ont un impact négatif sur la vie quotidienne de la femme et peuvent être atténués par certains par certaines stratégies telle que l’hystérectomie vaginale [5]. La maladie a une influence sur les attitudes et les comportements; les représentations de la maladie destructrice ou libératrice induisent des comportements: refus des soins et de recours au médecin dans le casde la maladie destructrice; rupture avec les contraintes sociales, enrichissement sur le plan personnel, lorsque la maladie est vécue sur le mode d´une libération. Il est question pour les femmes de croire à la disponibilité des ressources et opportunités individuelles pour pouvoir améliorer la qualité de vie et éviter les complications post opératoires.

Notre étude a montré que toutes les femmes étaient soulagées de leurs malaises; mais pour certaines, d’autres symptômes sont apparus liés aux complications postopératoires, telles queles douleurs, la plaie rectale, les dyspareunies; car il n’existe pas de chirurgie anodine. En effet, selon Hasson [6], la perte de l’organe prive les femmes des menstruations et peut être vécue par certaines comme une perte de l’identité, de la désirabilité malgré le fait que l’hystérectomie vaginale leur a permis d’être soulagé de douleurs ou de métrorragies invalidantes. Il y a peu de complications avec la voie vaginale, en effet, les femmes rapportaient peu de saignement en post opératoire. D’autres auteurs ont fait le même constat [7, 8]. Les dyspareunies transitoires à la reprise des rapports après hystérectomie vaginale ont été fréquentes. La pathogénie de ces dyspareunies selon Jewett [9] est le raccourcissement du vagin secondaire à la chirurgie, d’autres auteurs observent leur disparition à un an de la chirurgie [10].

Le vécu psychologique avant l’hystérectomie vaginale, est marqué par l’insatisfaction dans le désir sexuel, la perte de la féminité et la modification de l’estime de soi en raison des saignements du fibrome et du désagrément esthétique lié au prolapsus, incitaient les femmes à braver la peur de la chirurgie. A cela, s’ajoutaient les difficultés financières liées au coût de la chirurgie, engendrant le plus souvent l’insomnie. Dans la préparation psychologique préopératoire des patientes, souvent il manquait les informations sur les complications per et postopératoires. Selon Estrade et al [11], il faut savoir écouteravant de décider de toute chirurgie dans le cadre d’un consentement éclairé.

Le suivi postopératoire doit être assuré par le chirurgien lui-même pour compléter certaines informations, pour rassurer et accompagner la patiente. Ces quelques conseils simples peuvent minimiser les risques de décompensation psychique postopératoire. En cas des troubles sexuels, il faut s’interroger sur l’état psychologique pré opératoire des patientes et sur leur attente de cette chirurgie. D’après Soulier [12], les maladies répondent à des codes qui étaient pressentis depuis la nuit des temps dans tous les grands mythes et les grandes symboliques. Les recherches psychologiques récentes font qu´ils sont de mieux en mieux connus. Les dernières découvertes scientifiques viennent confirmer chaque jour ces compréhensions. Nous vivons dans un « Système de croyances », qui est propre à chacun et qui forme notre « vision du monde ». Ce système est différent du réel, mais nous pensons que c´est la réalité. Ce sont nos croyances. La maladie peut aujourd´hui se concevoir comme la conjonction d´un code de survie d´espèce et de la conséquence d´une croyance.

Nos patientes étaient mariées, et mère d’au moins deux enfants, en période de péri- ménopause, le plus souvent, la procréation pour elles, n’était plus une grande préoccupation, il en était de même pour l’absence de l’utérus causé par l’hystérectomie vaginale. Cependant les femmes éprouvent un sentiment de manque, de perte, elles ont l’impression qu’on leur a enlevé une partie de leur féminité et sombrent souvent dans la dépression. Même si le désir d’enfant n’existe plus, accepter l’incapacité physique de procréer est difficile pour certaines. D’autres percevront cette intervention comme une atteinte à leur féminité. Pour ces raisons il est indispensable que l’hystérectomie (totale ou non), soit le choix de la patiente et non pas seulement celui de l’équipe médicale [13]. Ainsi, selon Cailhier [14], pour favoriser des soins plus humains, il faut répondre à un besoin d´information tant sur la nature de la maladie que sur les traitements. Pour être utile, cette information doit répondre à un besoin identifié chez la patiente. L´investigation des besoins d´apprendre s´affirme comme étant une stratégie pour susciter la participation, l´intérêt et la prise en charge de la patiente. Le concept d’estime de soi, englobe l’image corporelle, l’idéal de soi, l’exercice de son rôle et l’identité personnelle [13]. L’image corporelle est l’image de notre corps que nous formons dans notre esprit, autrement dit, la façon dont notre corps nous apparait à nous même. C’est la somme de jugements conscients et inconscients que nous portons à l’égard de notre corps. Elle englobe les perceptions présentes et passées [15].

Nos patientes n’informaient ni les proches parents, ni leur entourage élargi sur leur état morbide, mais préféraient l’effet de surprise, juste après l’intervention. Cela est certainement un problème culturel, faisant penser que la médiatisation de la maladie fait courir le risque d’avoir un mauvais sort en per ou post-opératoire. Souvent, le statut socioculturel particulièrement important des maladies de la sphère urogénitale, les symptômes de la maladie principale, appartenant à la catégorie de ceux dont il est gênant d’en parler ou de s’en plaindre et qu’il vaut mieux cacher, en raison d’un fort sentiment de honte. De plus, se pose la question de l’incertitude concernant l’avenir des relations familiales et sociales. Les patientes ont présenté des doutes sur la façon dont elles allaient être acceptées «ainsi». Les membres de la société, n’étant pas tenu au courant de la maladie avant l’intervention, ne pouvaient aborder ce problèmeavec la patiente, après l’hystérectomievaginale. Le fait que dans la plupart des cas, la prise en charge globale de la maladie est le fait de mutualisation des efforts dans la société, cela peut avoir un impact négatif sur la santé des femmes, ayant subi une hystérectomie vaginale.

Tenant compte des symptômes gênant l’activité sexuel, pendant la maladie, la longue durée de prescription médicale de repos sexuel, post hystérectomie (environ 90 jours); des cas d’infidélité du conjoint, et même de non respect de la prescription médicale ont été notés. En effet, pour certains auteurs [8], le désir sexuel est un concept complexe, difficile à identifier et donc à analyser. Il est déterminé par l’interaction de la pulsion, des valeurs et des motivations.

## Conclusion

La maladie, par sa gravité potentielle, déstabilise non seulement la malade, mais également ses proches ; souvent les relations professionnelles sont plus ou moins interrompues. Les troubles psycho-sexuels, aggravés par l’atteinte de l’image corporelle pendant la maladie et le confort de la patiente, sont nettement améliorés par l’hystérectomie vaginale en raison aussi de l’absence « virtuelle » de cicatrice abdominale.

### Etat des connaissances actuelles sur le sujet

- La reprise des rapports sexuels est autorisée à la patiente 8 semaines après l’intervention;
- La prise en charge des couts des soins dans les pays à faible revenus est souvent le fait de la mutualisation des efforts dans la société.

### Contribution de notre étude à la connaissance

- Le risque de dyspareunie est très important après l’intervention; ce qui implique que 8 semaines d’autorisation de reprise de rapports sexuels paraissent trop précoces;
- La hantise de parler de la maladie aux parents et aux membres de la société est un handicap dans la prise en charge effective;
- L’incitation des praticiens à une bonne psychothérapie avant l’intervention est une assurance qualité de soins.
